# Identifying and understanding cognitive profiles in multiple sclerosis: a role for visuospatial memory functioning

**DOI:** 10.1007/s00415-024-12227-1

**Published:** 2024-02-26

**Authors:** Maureen van Dam, Eva A. Krijnen, Ilse M. Nauta, Tom A. Fuchs, Brigit A. de Jong, Martin Klein, Karin van der Hiele, Menno M. Schoonheim, Hanneke E. Hulst

**Affiliations:** 1 MS Center Amsterdam, Anatomy and Neurosciences, Vrije Universiteit Amsterdam, Amsterdam Neuroscience, Amsterdam UMC Location VUmc, Amsterdam, The Netherlands; 2 Institute of Psychology, Health, Medical and Neuropsychology Unit, Leiden University, Wassenaarseweg 52, Leiden, The Netherlands; 3 Department of Neurology, Massachusetts General Hospital, Harvard Medical School, Boston, MA USA; 4 MS Center Amsterdam, Neurology, Vrije Universiteit Amsterdam, Amsterdam Neuroscience, Amsterdam UMC Location VUmc, Amsterdam, The Netherlands; 5 Medical Psychology, Amsterdam UMC Location Vrije Universiteit Amsterdam, De Boelelaan 1117, Amsterdam, The Netherlands; 6 Leiden Institute for Brain and Cognition, Leiden University, Wassenaarseweg 52, 2333AK Leiden, The Netherlands

**Keywords:** Cognition, Profiles, Phenotypes, Classification, Multiple sclerosis, Latent profile analysis

## Abstract

**Background:**

The heterogeneous nature of cognitive impairment in people with multiple sclerosis (PwMS) hampers understanding of the underlying mechanisms and developing patient-tailored interventions. We aim to identify and classify cognitive profiles in PwMS, comparing these to cognitive status (preserved versus impaired).

**Methods:**

We included 1213 PwMS (72% female, age 45.4 ± 10.7 years, 83% relapsing–remitting MS). Cognitive test scores were converted to *Z*-scores compared to healthy controls for the functions: attention, inhibition, information processing speed (IPS), verbal fluency and verbal/visuospatial memory. Concerning cognitive status, impaired cognition (CI) was defined as performing at *Z* ≤ − 1.5 SD on ≥ 2 functions. Cognitive profiles were constructed using latent profile analysis on all cognitive functions. Cognitive profiles or status was classified using gradient boosting decision trees, providing the importance of each feature (demographics, clinical, cognitive and psychological functioning) for the overall classification.

**Results:**

Six profiles were identified, showing variations in overall performance and specific deficits (attention, inhibition, IPS, verbal fluency, verbal memory and visuospatial memory). Across the profiles, IPS was the most impaired function (%CI most preserved profile, Profile 1 = 22.4%; %CI most impaired profile, Profile 6 = 76.6%). Cognitive impairment varied from 11.8% in Profile 1 to 95.3% in Profile 6. Of all cognitive functions, visuospatial memory was most important in classifying profiles and IPS the least (area under the curve (AUC) = 0.910). For cognitive status, IPS was the most important classifier (AUC = 0.997).

**Conclusions:**

This study demonstrated that cognitive heterogeneity in MS reflects a continuum of cognitive severity, distinguishable by distinct cognitive profiles, primarily explained by variations in visuospatial memory functioning.

**Supplementary Information:**

The online version contains supplementary material available at 10.1007/s00415-024-12227-1.

## Introduction

The heterogeneous distribution of multiple sclerosis (MS)-related pathology gives rise to a variety of symptoms, including cognitive impairment [1, 2]. Cognitive impairment, characterized by impaired information processing speed (IPS), and verbal and visuospatial memory [2], substantially impacts daily functioning, work participation and ultimately quality of life [3]. Cognitive function in people with MS (PwMS) is assessed by neuropsychological examination using predefined test batteries [4], offering relevant information on whether an individual suffers from cognitive impairment, i.e., being “cognitively impaired” (CI) or “cognitively preserved” (CP). This dichotomization is often used in research. However, as individually affected domains can vary, a more detailed cognitive classification of PwMS could additionally allow health care providers to better tailor their treatment and offer more specific advise during a consultation, directing personalized medicine and tailored cognitive interventions [5].

Another way to enhance understanding of individuals’ cognitive performance is to identify “cognitive profiles”, e.g., by using latent profile analysis (LPA) [6]. LPA groups individuals into profiles based on specific characteristics in a data-driven manner [7]. Initial classification attempts in PwMS yielded multiple cognitive profiles [6, 8] and introduced the potential of staging and stratifying cognition in MS [9]. Depending on whether cognitive tests were used solely to characterize profiles or together with patient-reported outcome measures (e.g., mood), a different set of cognitive profiles emerged, i.e., five [6] versus four profiles [8], respectively. Interestingly, profiles could be ordered from preserved to impaired, possibly hinting toward a cognitive severity continuum. However, it remains unclear to what extent these profiles follow a single continuum or represent unique trajectories of cognitive impairment in PwMS. As well, the degree to which individual characteristics, e.g., mood or fatigue, are illustrative for the identified cognitive profiles and the contribution of these characteristics to distinguish between relevant profiles has not been studied before. Also, whether these profiles offer additional valuable insights beyond cognitive status (i.e., CP versus CI) is yet to be explored.

Therefore, the current study has four primary objectives: (1) to identify cognitive profiles in PwMS based on their cognitive performance, (2) to investigate the variability of demographic, clinical, and psychological factors (anxiety, mood, and fatigue) among the found profiles, (3) to assess which characteristics contribute the most to distinguishing the cognitive profiles, and (4) to evaluate whether determining cognitive profiles offers additional information on cognition beyond cognitive status (i.e., CP versus CI).

## Methods

### Study population and design

The study retrospectively evaluated cross-sectional data from ten observational studies conducted between 2008 and 2023 at the Amsterdam UMC location VUmc and 16 outpatient MS clinics across the Netherlands [10–16]. Data of PwMS were included if they had a clinically definite diagnosis of MS or clinically isolated syndrome, along with available neuropsychological and neurological assessment, and questionnaire data. Supplementary Table 1 summarizes cohort details and inclusion/exclusion criteria. If PwMS participated in multiple studies or visits (*n* = 43), only their initial visit was included, resulting in a total of 1213 PwMS eligible for subsequent analysis.

### Ethical standards statement

Ethical approval for the studies was granted by the Medical Ethics Review Committee of Amsterdam UMC and the Medical Ethical Committee Brabant University. All included PwMS provided written informed consent.

### Demographics and clinical and psychological functioning

Demographic characteristics included age, sex, and level of education (according to the Verhage classification) [17]. MS type was based on relapsing–remitting MS (RRMS), primary and secondary progressive MS (PPMS, SPMS), clinically isolated syndrome (CIS), and unknown. Disease duration was based on date of diagnosis. Physical disability was based on the Expanded Disability Status Scale (EDSS) score, which was collected by a certified examiner either physically during consultation or via a validated telephone version [18, 19]. Anxiety and depression symptoms were measured using the Hospital Anxiety and Depression Scale (HADS) [20], and levels of fatigue using the Checklist Individual Strength-20 revised (CIS20-R) [21].

### Neuropsychological examination

Cognitive functioning was assessed using Dutch adaptations of the Minimal Assessment of Cognitive Function in MS (MACFIMS) [22] and/or the Brief Repeatable Battery of Neuropsychological tests (BRB-N) [23]. As different tests were used in different cohorts, cognitive test scores were averaged and analyzed using the following cognitive functions: attention, inhibition (subdomain of executive functioning (EF)), IPS, verbal fluency (subdomain of EF), verbal memory, and visuospatial memory. Cognitive test scores corresponding to cognitive functions are detailed in Supplementary Table 2. All scores were corrected for healthy control effects in age, sex and educational level, and transformed into function-specific Z-scores relative to controls, based on normative data (*n* = 407). If a PwMS scored below − 1.5 standard deviations (SD) on a cognitive function, that function was considered impaired. If this criterion was met for at least two cognitive functions, the PwMS was categorized as having cognitive impairment (CI) [24]. Otherwise, PwMS were classified as cognitively preserved (CP).

### Statistical analyses

#### Cognitive profile identification

Analyses were conducted in SPSS 28.0 (IBM, Armonk, NY, USA) and R-Studio (v4.2.1) [25]. Cognitive profiles were identified using latent profile analysis (LPA) based on the continuous cognitive Z-scores [26]. LPA is based on probability theory and clusters in a person-centered way (using characteristics of individuals). Unlike K-means clustering, LPA uses model fit statistics to determine the optimal number of profiles, eliminating a priori cluster number specification [27]. The ‘Mclust’ algorithm (‘tidyLPA’ package) was used for LPA [28]. The model was specified under the assumption of varying variances and covariances of included variables, estimating a range of two to six profiles (based on prior research on cognitive profiles in MS) [6]. The missing data rate for each variable was < 9%, except for fatigue (40.4%; Supplementary Table 3). Missing values were imputed using the ‘MissForest’ package, utilizing demographics, clinical, psychological and cognitive variables, which is a random forest algorithm providing non-parametric missing value imputation [29]. This algorithm has been shown to outperform other imputation strategies, particularly in case of mixed-type data [30]. Supplementary Table 3 describes the sample using imputed and non-imputed data. We assessed the model fit using the Akaike information criterion and Bayesian information criterion, with lower values indicating a better fit [31]. We assessed classification accuracy, targeting an average posterior class probability of ≥ 0.70 for each profile (reflecting mean probability of belonging to a profile) and a classification reliability coefficient (entropy values) of ≥ 0.60 [31]. PwMS were assigned to the profile with the highest probability of profile membership, thereby reflecting patterns of cognitive performance rather than significant differences between profiles on each individual cognitive function.

#### Profile characterization

Differences between cognitive profiles on demographic, clinical, psychological, and cognitive functioning were tested using multivariate linear models for continuous variables and Chi-square tests for categorical variables. Post hoc tests, adjusted for multiple comparisons using Bonferroni, assessed profile differences. An *α*-level of 0.05 was considered statistically significant.

#### Classifying cognitive profiles and status

Classifications trees were built to classify cognitive profiles based on available characteristics and to assess whether these profiles offer additional information beyond cognitive status. We constructed three classification models per outcome measure (outcome measures: cognitive profiles or status): (1) using only cognitive functions as features, (2) demographics and clinical functioning as features, and (3) demographics and clinical and psychological functioning as features. Using gradient boosting decision trees (‘xgboost’ package) [32], we were able to evaluate the contribution of each feature, henceforth referred to as ‘importance’ [33]. For gradient boosting, data was divided into a train (60%) and a test (40%) set. Due to small sample sizes in some profiles, splitting the training data into a validation set was not possible, which would be the preferred strategy. To balance the profiles in both datasets, we used profile classification as stratification factor (leading to an equal number of PwMS per profile in each dataset). Tuning of hyper-parameters of the model was done by applying a grid search to avoid overfitting (Supplementary Table 8 details the parameters). Model performance was evaluated using the area under the curve (AUC): an AUC of 0.6–0.7 was considered ‘poor’, but ‘acceptable’ from 0.7 onward [34]. Following the decision trees, we analyzed feature importance scores, which indicate each feature’s utility in constructing the decision tree. Higher importance reflects greater involvement in key decisions, providing a feature ranking within the model.

## Results

### Patients’ descriptives

The sample included 1213 PwMS (Table 1; 71.89% female, mean age 45.39 ± 10.67 years, median educational level 6 (“finished high level secondary education”), median EDSS 3.00 (interquartile range 2.00–4.00), mean disease duration 9.84 ± 7.68 years). The distribution of MS types included: 82.90% RRMS, 10.06% SPMS, 5.19% PPMS, 1.48% PwMS with an unknown type, and 0.41% CIS.

**Table 1:** An overview of the demographics, clinical functioning, cognitive functioning. and PROMS per cognitive profile

	The sample (*n* = 1213)	Profile 1 (*n* = 85)	Profile 2 (*n* = 277)	Profile 3 (*n* = 41)	Profile 4 (*n* = 332)	Profile 5 (*n* = 371)	Profile 6 (*n* = 107)	*p*^b^
*Demographics*								
Sex|female (%)	872 (71.9%)	66 (77.6%)	214 (77.3%)	32 (78.0%)	217 (65.4%)	267 (72.0%)	76 (71.0%)	0.066
Age	45.4 ± 10.7	43.9 ± 10.4	43.3 ± 9.8	45.5 ± 10.3	45.9 ± 10.8	45.9 ± 10.9	48.8 ± 11.1	< 0.001*
Education^a^	6.0 (5.0–6.0)	6.0 (5.0–6.0)	6.0 (5.0–6.0)	5.0 (5.0–6.0)	6.0 (5.0–6.0)	6.0 (5.0–6.0)	6.0 (5.0–6.0)	1.000
*Clinical functioning*								
MS type|*n* (%)								< 0.001*
RRMS	1005 (82.9%)	76 (89.4%)	259 (93.5%)	37 (90.2%)	267 (80.4%)	290 (78.2%)	76 (71.0%)	
SPMS	122 (10.1%)	8 (9.4%)	10 (3.6%)	4 (9.8%)	33 (9.9%)	48 (12.9%)	19 (17.8%)	
PPMS	63 (5.2%)	1 (1.2%)	5 (1.8%)	0 (0%)	22 (6.6%)	28 (7.5%)	7 (6.5%)	
CIS	5 (0.4%)	0 (0%)	1 (0.4%)	0 (0%)	3 (0.9%)	0 (0%)	1 (0.9%)	
Unknown	18 (1.5%)	0 (0%)	2 (0.7%)	0 (0%)	7 (2.1%)	5 (1.3%)	4 (3.7%)	
Disease duration	9.8 ± 7.7	8.9 ± 7.4	8.3 ± 6.7	10.2 ± 7.8	7.5 ± 6.2	10.2 ± 7.8	13.1 ± 9.0	< 0.001*
EDSS	3.3 ± 1.6	3.2 ± 1.7	2.7 ± 1.5	3.5 ± 1.6	3.3 ± 1.3	3.5 ± 1.6	4.2 ± 1.5	< 0.001*
*Cognitive functioning*								
Cognitive status | CI (%)	402 (33.1%)	10 (11.8%)	40 (14.4%)	8 (19.5%)	111 (33.4%)	131 (35.3%)	102 (95.3%)	< 0.001*
Attention | CI (%)	− 0.8 ± 1.2 (22.8%)	− 0.4 ± 0.7 (5.9%)	− 0.1 ± 0.7 (4.0%)	− 0.5 ± 0.3 (0.0%)	− 0.4 ± 0.9 (13.6%)	− 1.1 ± 1.1 (35.6%)	− 2.6 ± 1.6 (78.5%)	< 0.001*
Inhibition | CI (%)	− 0.5 ± 1.3 (19.0%)	0.2 ± 1.1 (8.2%)	0.1 ± 0.8 (1.8%)	− 0.6 ± 0.6 (9.8%)	− 0.3 ± 1.2 (16.0%)	− 0.9 ± 1.1 (27.8%)	− 1.8 ± 2.0 (55.1%)	< 0.001*
IPS | CI (%)	− 1.1 ± 1.2 (34.2%)	− 0.5 ± 1.4 (22.4%)	− 1.0 ± 0.8 (27.1%)	− 1.1 ± 0.6 (19.5%)	− 0.9 ± 1.1 (28.6%)	− 1.2 ± 1.3 (36.7%)	− 2.4 ± 1.1 (76.6%)	< 0.001*
Verbal fluency | CI (%)	− 0.7 ± 0.8 (12.2%)	− 0.3 ± 0.8 (7.1%)	− 0.3 ± 1.0 (11.2%)	− 0.7 ± 0.4 (0.0%)	− 0.5 ± 0.6 (6.6%)	− 1.1 ± 0.4 (14.3%)	− 1.2 ± 0.8 (33.6%)	< 0.001*
Verbal memory | CI (%)	− 0.7 ± 1.2 (22.8%)	− 0.3 ± 0.7 (3.5%)	− 0.8 ± 1.1 (27.1%)	− 0.7 ± 0.4 (7.3%)	− 0.6 ± 1.2 (22.9%)	− 0.3 ± 0.8 (10.0%)	− 2.3 ± 1.3 (76.6%)	< 0.001*
Visuospatial memory | CI (%)	− 0.4 ± 1.0 (14.6%)	− 0.4 ± 0.4 (1.2%)	0.1 ± 0.3 (0.0%)	− 0.6 ± 0.9 (17.1%)	− 0.9 ± 1.3 (33.1%)	− 0.1 ± 0.8 (3.5%)	− 1.4 ± 1.1 (43.0%)	< 0.001*
*Psychological functioning*								
HADS-A	6.3 ± 3.7	5.5 ± 3.4	6.5 ± 3.3	6.6 ± 3.8	6.0 ± 3.5	6.3 ± 3.9	7.0 ± 4.4	0.180
HADS-D	4.6 ± 3.6	3.7 ± 3.4	4.1 ± 3.1	5.7 ± 3.9	4.4 ± 3.4	5.1 ± 4.0	5.6 ± 3.4	< 0.001*
CIS20-R	79.7 ± 21.1	72.2 ± 21.6	76.2 ± 17.4	84.6 ± 19.0	78.1 ± 21.7	82.6 ± 22.1	88.1 ± 20.7	< 0.001*

Displayed are the mean ± standard deviation

Abbreviations: *RRMS* relapsing–remitting MS,* SPMS* secondary progressive MS, *PPMS* primary progressive MS, *CIS* clinically isolated syndrome, *EDSS* Expanded Disability Status Scale,* CI* cognitively impaired, *IPS* information processing speed, *PROMS* patient-reported outcome measures, *HADS-A* Hospital Anxiety and Depression Scale (HADS)-Anxiety subscale, *HADS-D* HADS-Depression subscale,* CIS20-R* Checklist Individual Strength 20-Revised

^a^For ordinal or not normally distributed variables, median and (interquartile range) are displayed

^b^Corrected *p* values

*Significant between all profiles, at an α-level of 0.05, after correcting for multiple comparisons using Bonferroni

### Identification of cognitive profiles

**Fig. 1:**
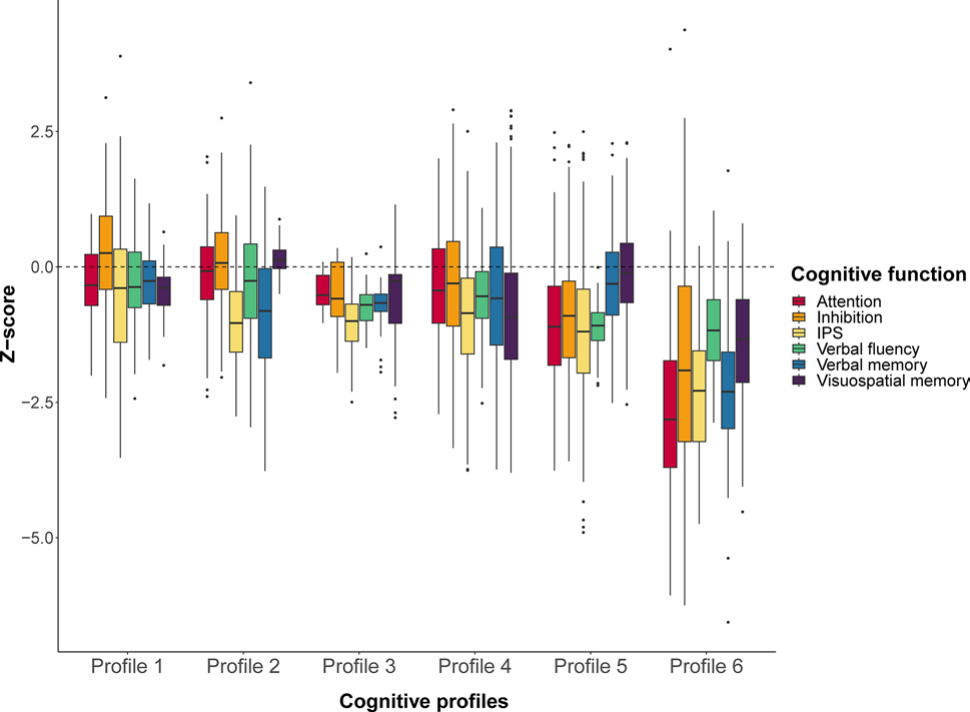
Depiction of the cognitive functions (Z-scores) per cognitive profile. On the y-axis, the dashed line indicates average performance at Z = 0.0. Abbreviation:* IPS* information processing speed

**Fig. 2:**
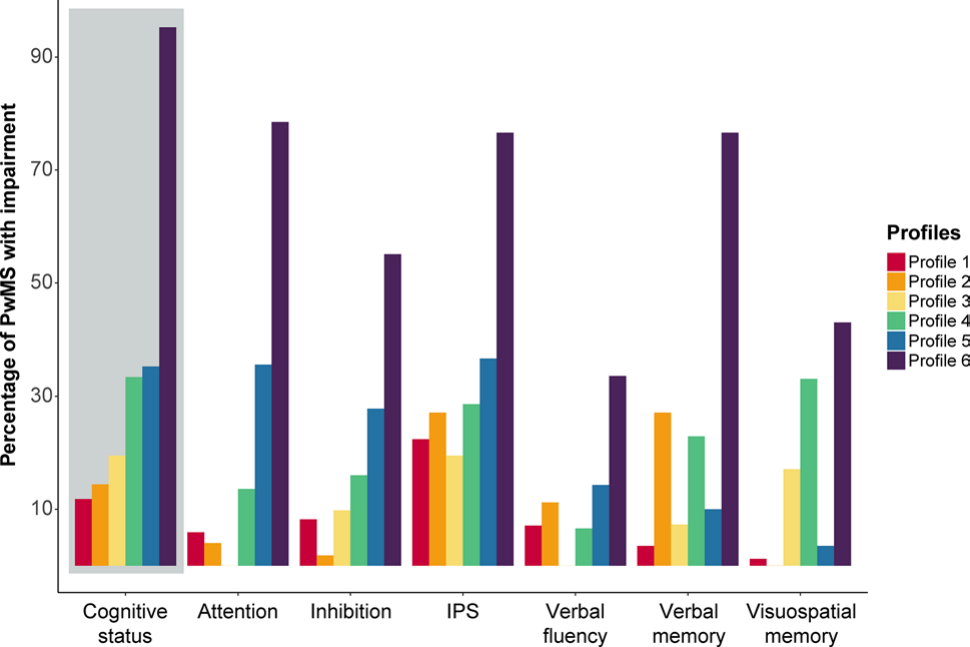
The percentage of PwMS with cognitive impairment per cognitive functions or classified as cognitively impaired (cognitive status). Abbreviations: *IPS* information processing speed, *PwMS* people with MS

**Fig. 3:**
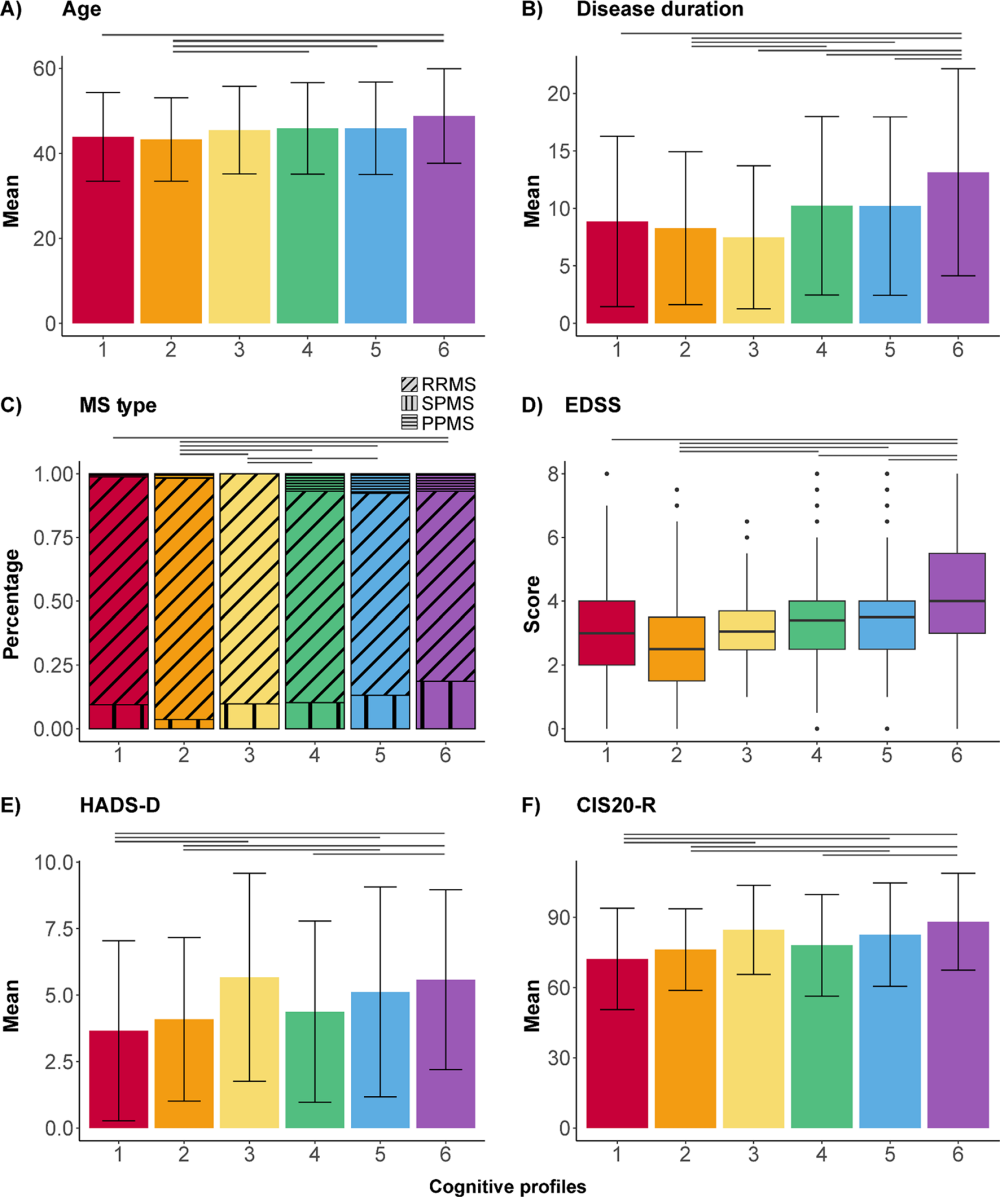
Significant post hoc differences (indicated with a black stripe) between cognitive profiles (number on the x-axis) for age (**A**), disease duration (**B**), MS type (**C**), physical disability (EDSS; **D**), symptoms of depression (HADS-D; **E**), and level of fatigue (CIS20-R; **F**). Abbreviations: *RRMS* relapsing–remitting MS, *SPMS* secondary progressive MS, *PPMS* primary progressive MS,* EDSS* Expanded Disability Status Scale,* HADS-D* Hospital Anxiety and Depression Scale (HADS)-Depression subscale, *CIS20-R* Checklist Individual Strength 20-Revised

LPA identified six cognitive profiles (Fig. 1), showing the best model fit and an appropriate classification accuracy (classification reliability coefficient = 0.61; average posterior class probabilities ranged between 0.66 and 0.83, with Profile 1 being the lowest and Profile 3 the highest; Supplementary Fig. 1). Visual inspection revealed an even distribution of PwMS from each cohort across the six profiles (Supplementary Fig. 2). All profiles are described in Table 1 (non-imputed data in Supplementary Table 4). The distribution of impaired cognitive functions and cognitive status is illustrated in Fig. 2 (post hoc differences are included in Supplementary Table 5). Supplementary Fig. 3 highlights significant post hoc differences in cognitive functions between profiles, while Fig. 3 illustrates differences in demographics and clinical and psychological functioning. A brief summary of each profile is provided below.

***Profile 1.*** In Profile 1 (*n* = 85), most PwMS showed preserved performance across all functions, with the only notable lower performance in IPS (Z-score = − 0.54 ± 1.35, impaired in 22.35%). This profile had fewer PwMS with CI (11.76%), compared to Profiles 4–6 (*p* values < 0.001) and showed limited symptoms of depression (mean = 3.66 ± 3.39) and fatigue (mean = 72.20 ± 21.62; range *p* values ≤ 0.001–0.042).

***Profile 2.*** In Profile 2 (*n* = 277), most PwMS demonstrated preserved performance in attention, inhibition, verbal fluency, and visuospatial memory (range Z-scores = 0.14 to − 0.26). Notably, 27.08% of PwMS displayed impairment in IPS and verbal memory, while 11.19% showed an impaired verbal fluency. This profile had fewer PwMS with CI (14.44%) than Profiles 4–6 (*p* values < 0.001), marked by a relatively young age, short disease duration, low EDSS, and a high proportion of RRMS (93.50%, highest of all profiles).

***Profile 3.*** In Profile 3 (*n* = 41), most PwMS displayed preserved performance across all functions (range Z-scores = − 0.47 to − 0.74), with the lowest Z-score in IPS (Z-score = − 1.05 ± 0.63). This profile included 19.51% PwMS with an IPS impairment, while 17.07% showed impairment in visuospatial memory. This profile consisted of fewer PwMS with CI (19.51%), compared to Profile 5 (*p* = 0.042) and 6 (*p* < 0.001). The relatively high proportion of RRMS (90.24%) and the high scores on depression (mean = 5.67 ± 3.91) and fatigue (mean = 84.61 ± 19.01) were most characteristic (range *p* values ≤ 0.001–0.049).

***Profile 4.*** In Profile 4 (*n* = 332), most PwMS showed preserved performance in inhibition, attention, and verbal fluency (range Z-scores = − 0.27 to − 0.47). Visuospatial memory was impaired in 33.13% of PwMS, while IPS and verbal memory were impaired in 28.61% and 22.89%, respectively. This profile ranked fourth, due to its higher percentage of PwMS with CI (33.43%), compared to the better-performing profiles (*p* values < 0.001). Considering cognitive performance, age, disease duration and EDSS, this profile could be classified as an “in-between” profile (range *p values* ≤ 0.001–0.034). Profile 4 performed worse compared to Profile 5 on verbal (*p* < 0.001) and visuospatial memory (*p* = 0.016).

***Profile 5.*** In Profile 5 (*n* = 371), 36.66% of PwMS displayed an IPS impairment, 35.58% an attention impairment, 27.76% an inhibition impairment, and 14.29% a verbal fluency impairment (range Z-scores = − 0.91 to − 1.19). Visuospatial memory in Profile 5 was higher (Z-score = − 0.10 ± 0.79, %CI = 3.50%) compared to all profiles (range *p* values ≤ 0.001–0.027), except compared to Profile 2 (Z-score = 0.14 ± 0.25, *p* = 0.019). Although not as high as visuospatial memory, verbal memory was also relatively preserved in this profile (Z-score = − 0.33 ± 0.83, %CI = 9.97%). This profile consisted of more PwMS with CI (35.31%) compared to Profiles 1–3, but fewer compared to Profile 6 (range *p* values ≤ 0.001–0.042). This profile differed from Profile 6 in terms of a shorter disease duration (*p* = 0.002) and a lower EDSS (*p* = 0.003). Profile 5 had fewer RRMS PwMS (78.17%), a higher age and EDSS, a longer disease duration, and more symptoms of depression and fatigue compared to other profiles (range *p* values ≤ 0.001–0.021).

***Profile 6.*** In Profile 6 (*n* = 107), most PwMS were classified as being CI (95.33%), and performance was lower on all functions compared to other profiles, except for verbal fluency (Z-score = 1.16 ± 0.81, which was similar to Profile 5 (*p* = 1.000)). Percentages of impairment on function level ranged between 33.64% (for verbal fluency) to 78.50% (for attention). This profile had the lowest proportion of RRMS (71.03%). This profile was marked by an older age and worse performance on all clinical variables (range *p* values ≤ 0.001–0.022). Depression (mean = 5.58 ± 0.3.38) and fatigue (mean = 88.10 ± 20.65) were higher for Profile 6, compared to Profile 1, 2, and 3 (range *p* values ≤ 0.001–0.034).

### Classification of cognitive profiles

Cognitive profiles were classified based on cognitive functions, demographics, and clinical and psychological features. No differences were observed between train and test data for these variables (Supplementary Table 6). Table 2 summarizes model performance (AUC) for both datasets, along with feature importance.

**Table 2:** Results on the decision tree analyses, including the importance per feature in the model of the test data (ranking variable order from highest relative importance to lowest) and the area under the curve (AUC) per feature in the models of both the train and test dataset

	Importance		AUC	
	Relative importance	Rank of importance within the model	Train data	Test data
***Cognitive profiles***				
*Model 1*			0.969	0.910
Attention	0.181	2		
Inhibition	0.112	5		
IPS	0.053	6		
Verbal fluency	0.173	3		
Verbal memory	0.168	4		
Visuospatial memory	0.313	1		
*Model 2*			0.754	0.629
Sex	0.035	5		
Age	0.382	1		
Education	0.050	4		
MS type	0.026	6		
Disease duration	0.286	2		
EDSS	0.221	3		
*Model 3*			0.785	0.661
Sex	0.019	8		
Age	0.250	2		
Education	0.042	7		
MS type	0.011	9		
Disease duration	0.228	3		
EDSS	0.097	4		
HADS-A	0.049	6		
HADS-D	0.048	5		
CIS20-R	0.256	1		
***Cognitive status***				
*Model 1*			0.999	0.997
Attention	0.244	2		
Inhibition	0.112	4		
IPS	0.301	1		
Verbal fluency	0.064	6		
Verbal memory	0.173	3		
Visuospatial memory	0.107	5		
*Model 2*				
Sex	0.055	5	0.741	0.633
Age	0.172	3		
Education	0.056	4		
MS type	0.027	6		
Disease duration	0.373	1		
EDSS	0.317	2		
*Model 3*			0.789	0.646
Sex	0.031	8		
Age	0.174	3		
Education	0.048	7		
MS type	0.008	9		
Disease duration	0.301	1		
EDSS	0.177	2		
HADS-A	0.051	6		
HADS-D	0.087	5		
CIS20-R	0.122	4		

Abbreviations: *IPS* information processing speed, *EDSS* Expanded Disability Status Scale, *HADS-A* Hospital Anxiety and Depression Scale (HADS)-Anxiety subscale, *HADS-D* HADS-Depression subscale, *CIS20-R* Checklist Individual Strength 20-Revised

**Fig. 4:**
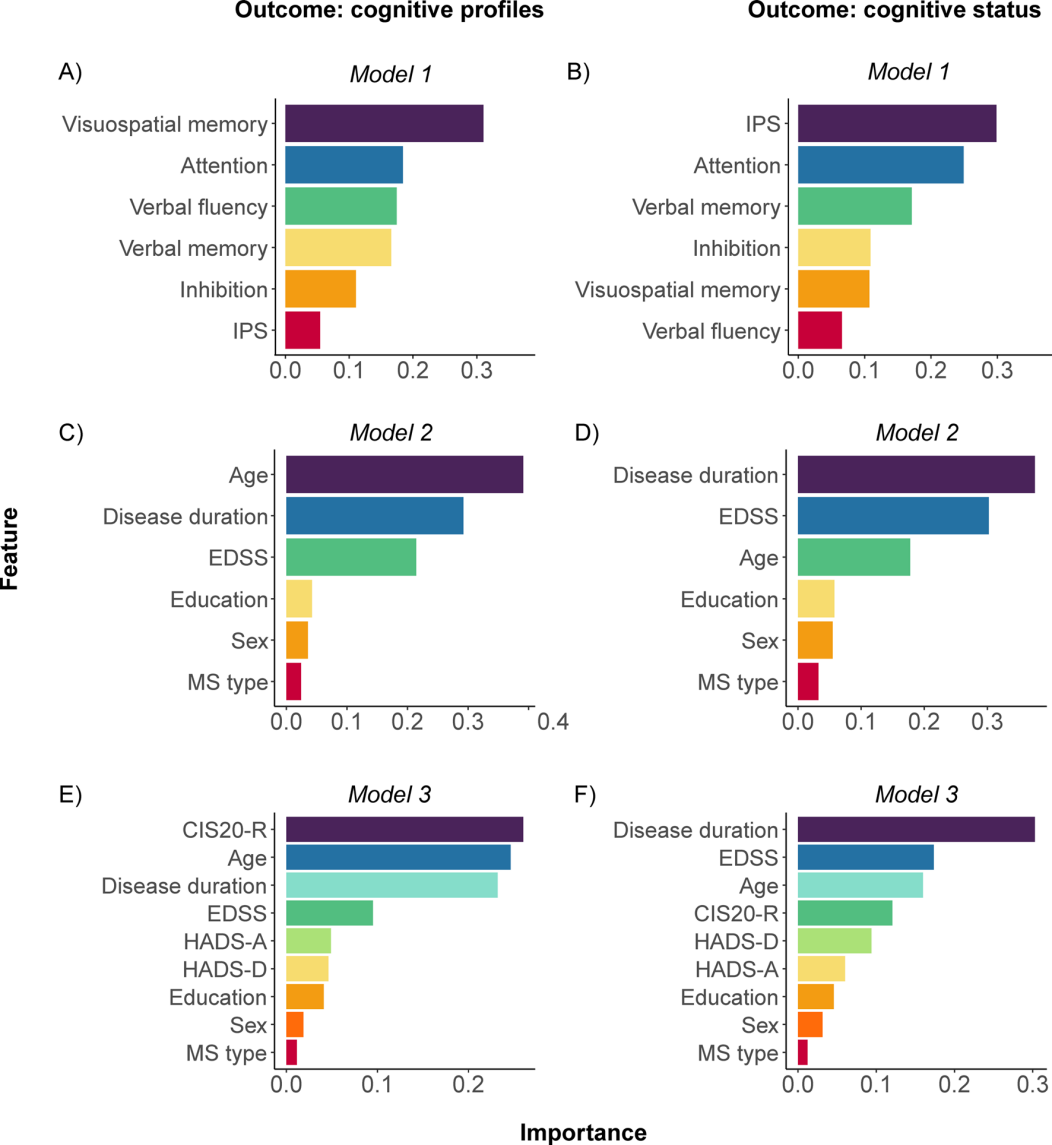
Depiction of the (relative) importance of each feature in the model, with either cognitive profiles or cognitive status as outcome measure. Panel A and B depict the use of cognitive functions as features (model 1). Panel C and D depict the use of demographics and clinical functioning as features (model 2). Panel E and F depict the use of demographics, clinical and psychological functioning as features (model 3). Abbreviations: *IPS* information processing speed,* EDSS* Expanded Disability Status Scale, *HADS-A* Hospital Anxiety and Depression Scale (HADS)-Anxiety subscale, *HADS-D* HADS-Depression subscale, *CIS20-R* Checklist Individual Strength 20-Revised

***Model 1: cognitive functions.*** Visuospatial memory was the most important feature in classifying cognitive profiles, while IPS was the least important (Fig. 4A, AUC = 0.910).

***Model 2: demographics and clinical functioning.*** Model 2 had substantially lower classification performance (AUC = 0.629) compared to model 1. Age was the most and MS type was the least important feature in the model (Fig. 4C).

***Model 3: demographics and clinical and psychological functioning.*** Classification performance improved slightly (AUC = 0.661) when adding psychological features to model 2. Fatigue and age were most important in classifying profiles, while MS type was the least important feature (Fig. 4E).

### Classification of cognitive status

We applied similar models to classify PwMS as CP or CI (Table 2 for model performance), with between-group differences regarding the features summarized in Supplementary Table 7.

***Model 1: cognitive functions.*** IPS was the most important feature in classifying cognitive status, with verbal fluency being the least important feature (Fig. 4B, AUC = 0.997).

***Model 2: demographics and clinical functioning.*** As to classifying profiles, using demographics and clinical features led to a similar drop in performance (AUC = 0.633) compared to model 1, when classifying cognitive status. Disease duration was the most important feature, while MS type was least important (Fig. 4D).

***Model 3: demographics and clinical and psychological functioning.*** A slight performance improvement was found when adding psychological features to model 2 (AUC = 0.646). Disease duration remained the most important feature, with MS type ranking the lowest (Fig. 4F).

## Discussion

In this retrospective cross-sectional study (*n* = 1213), we investigated the potential of phenotyping and classifying cognitive performance in MS. With LPA, we distinguished six cognitive profiles. PwMS within these six profiles differed in cognitive performance on specific domains, but also in clinical functioning (MS type, disease duration, and EDSS), mood, and fatigue. Interestingly, visuospatial memory was relatively most important in classifying these profiles and IPS the least. For cognitive status (cognitively impaired versus cognitively preserved), a concept widely used especially in research, IPS was the most important classifier. These findings emphasize the existence of different cognitive profiles in MS and their potential to provide additional information compared to the current standard, i.e., cognitive status.

Our study supports the notion of cognitive profiles in MS. Like other symptoms observed in PwMS, cognitive impairment is largely heterogeneous in prevalence and severity [1] and can manifest in various forms, primarily affecting domains such as IPS, and verbal and visuospatial memory [2]. Some PwMS experience cognitive impairment at disease onset, while others worsen over time [35]. This study aimed to enhance understanding of the prevalence and severity of cognitive dysfunctioning, by identifying and characterizing cognitive profiles using LPA. LPA offers the possibility to capture subtle changes in cognitive performance, as it is a fine-grained, “person-centered” method that can probabilistically group individuals with similar ‘cognitive’ configurations, and hence profiles, using a certain set of variables [36].

We identified six profiles, which differed on overall performance and specific deficits (attention, inhibition, IPS, verbal fluency, verbal memory and visuospatial memory). Across the profiles, IPS was the most impaired function (occurring in 22.4% of PwMS in the most preserved profile, up to 76.6% in the most impaired profile). In the literature, the number and description of these profiles vary from previously identified cognitive profiles in MS depending on the chosen strategy (theory-driven versus data-driven clustering methods) and input variables (cognitive tests alone versus together with questionnaires). In two prior studies, four profiles were identified theoretically, i.e., based on Z-scores interpretation from predefined domains [37, 38]. In a study similar to ours (*n* = 1212), data-driven LPA yielded five cognitive profiles instead of six [6]. In comparison to the previous study, we identified similar profiles, including one with (relatively) preserved cognitive function, another displaying mild verbal memory and verbal fluency deficits, a profile marked by severe attention and executive functioning, and a profile characterized by severe impairments in multiple cognitive domains. Contrarily, the previous study identified a profile described as mild-multidomain, which included mild impairments in verbal memory, attention/inhibition, and IPS. In our cohort, this mild-multidomain profile would be described as displaying mild impairment in IPS and visuospatial memory. Furthermore, we were also able to identify a profile with severe visuospatial memory performance, alongside mild IPS and verbal memory performance. Several factors may account for these study differences, including variations in: (1) the construction of cognitive functions as input variables (e.g., using single tests versus multiple tests, or averaging subscales versus using subscales separately), (2) the range of cognitive performance in the sample (our sample had less variability, potentially due to test averaging), (3) the actual test performance (our sample had fewer difficulties in verbal fluency, but more in memory, particularly visuospatial memory), and (4) the sample selection. Cross-cultural differences may have influenced cognitive performance, which has been increasingly recognized as challenging in neuropsychological testing [39]. This underscores the importance of careful consideration in future research and the improvement of normative data. We considered the impact of the patient sample selection minimal, given the overlapping selection procedures (retrospectively combining multi-center data), similar inclusion/exclusion criteria, and comparable demographics and clinical functioning. As such, cognitive profiles can be identified when combining multiple cohorts with varying cognitive tests, which is particularly promising in a clinical context that often involves various test evaluations. Ensuring international replicability of these profiles is a crucial focus for future research.

When classifying PwMS into cognitive profiles, *memory functioning* was particularly relevant in classifying PwMS into cognitive profiles compared to IPS in cognitive status. Surprisingly, in profile classification, visuospatial memory was ranked most important and IPS the least. Conversely, for cognitive status, IPS appeared the most important feature. This aligns with prior research, highlighting IPS as the most sensitive function for detecting and monitoring cognitive impairment in MS, as it underlies, or at least supports, multiple cognitive processes [2, 40]. Additionally, the finding that IPS was fairly impaired even within the least affected cognitive profile fits with the concept of IPS being the initial impairment in the early stages of the disease [41], preceding impairments in other domains [42]. Despite their high sensitivity, tests used to assess IPS functioning have been criticized for their lack of specificity, i.e., IPS being impaired in all PwMS [43]. Previously, it has been mentioned that in distinguishing cognitive impairment in PwMS from healthy controls, memory tests are nearly as effective as IPS tests [40]. Memory tests show only slightly lower effect sizes (with a mean Cohen’s *d* for the Symbol Digit Modalities Test measuring IPS at 1.11, while memory tests range between 1.03, 0.89, and 0.86), despite a greater variety in the tests used to measure memory function [40]. Both IPS and memory impairments are highly prevalent, with IPS difficulties reported in 40–70% of the PwMS [44], and memory difficulties reported in 40–65% of PwMS [45]. Building on recent research suggesting that memory impairments may develop following deficits in IPS and learning [42], we propose a significant role for memory functioning, especially visuospatial memory, in capturing part of the cognitive heterogeneity in MS. In particular, visuospatial memory displayed the most pronounced differences between the cognitive profiles, suggesting its specificity for assessing cognitive functioning in MS over IPS. Notably, we observed that even in a more impaired profile, memory function was, on average, preserved. These findings align with the approach of screening tools, such as the Brief International Cognitive Assessment for MS [46], which assess memory function, rather than relying solely on IPS.

In this study, the profiles differed not only in cognitive functioning, but also in age, MS type, disease duration, EDSS, and mood and fatigue (although mean differences between the profiles appeared subtle). Previous studies offered limited insights into variations in demographical, clinical, and psychological characteristics. One study proposed a *continuum* where the severity increases as the profiles worsen [8]. Indeed, when examining cognitive profiles, they often appear to result from a linear severity continuum, a significant observation also raised in other diseases such as schizophrenia [47]. Hence, it was pivotal to ascertain that current profiles displayed unique configurations. Fatigue specifically played a potentially important role in classifying cognitive profiles. Fatigue and cognitive functioning have been found to show a complex interrelationship in MS [48], although it remains currently unknown how both factors affect each other [35]. It is noteworthy to mention that here, reports on fatigue were available for only 60% of PwMS versus less than 9% for other variables, highlighting the need for careful interpretation. Considering the relatively low AUC of these classifications, suggesting an equal room for improvement when classifying cognitive profiles and status, it raises the question of whether radiological variables would add explained variance. In light of our classification results, additional efforts could explore generating profiles based on demographics and questionnaires, including fatigue, to gain further valuable insights [8]. Together, this suggests that the identified profiles represent a continuum rooted in the severity of cognitive impairment and can be distinguished through data-driven approaches to identify cognitive subtypes. These configurations, or profiles, hold promise to inform treatment and to tailor interventions. For instance, targeting memory functioning may not be recommended for PwMS with a high likelihood of belonging to Profile 5, while addressing depression might be suitable for those in Profile 3.

This study is not without limitations. Data-driven profiles prompt the question of their dependency on the cognitive functions used as input [36] as well as the choice of fit statistics [28]. The discrepancy in the literature stresses the need to replicate these profiles using alternative input strategies and evaluating established profiles on independent datasets. Furthermore, the current large sample comprised retrospective data from ten different cohorts, which is one of the strengths of the study. It also presented challenges in calculating cognitive functions. For instance, not all cohorts had information on working memory and/or cognitive flexibility (functions known to be affected in MS [1]). While our dataset did not allow for an investigation into the sub-aspects of overall cognitive function, such as precision or recall versus recognition, exploring these aspects is crucial for a more comprehensive understanding of cognitive profiles. Additionally, the limited absolute differences between profiles, attributed to sample characteristics such as a high number of RRMS and level of fatigue, limit our ability to draw conclusions about the clinical significance of profile differences and should therefore be carefully interpreted, possibly due to lower sample sizes in some profiles. The lower sample size in some of the profiles (with the lowest sample size being 41 for Profile 3, compared to the highest sample size of 371 for Profile 5) also requires careful interpretation. Constructing a validation sample was not feasible, which would be the preferred strategy to avoid overfitting. However, this limitation was somewhat mitigated by splitting the dataset into a large test dataset (40% of the data) and a training dataset (60% of the data). Additionally, a stratification factor was employed to ensure equal proportions of people in both the training and test datasets. Finally, cognitive profiles have not been studied longitudinally, limiting our understanding of their stability and their predictive value. Latent mixture modeling techniques can be extended to include changes over time or within-profile variations, guiding future research directions.

In conclusion, this study showed that cognitive heterogeneity in MS appears as a severity continuum of cognitive decline, distinguishable by cognitive profiles, primarily differentiated by visuospatial memory function. By identifying these profiles, our goal was to move toward tailoring treatments to the individual in the future and more precise monitoring of cognitive function in MS. Exploring the stability, replicability, and the profiles’ etiology are crucial for future research to facilitate their clinical application.

### Supplementary Information

Below is the link to the electronic supplementary material.

Supplementary file1 (DOCX 483 KB)

Supplementary file2 (DOCX 29 KB)

## Data Availability

Anonymized data can be shared upon reasonable request from a qualified investigator.
