# Supplementary material for: Identifying and understanding cognitive profiles in multiple sclerosis: a role for visuospatial memory functioning
Source: J Neurol. 2024 Feb 26;271(5):2195–206. doi: 10.1007/s00415-024-12227-1 (PMC11055708; doi:10.1007/s00415-024-12227-1)
Supplement: Supplementary file 1 — Supplementary file1 (DOCX 483 KB) [file 415_2024_12227_MOESM1_ESM.docx]

**Supplementary materials**

**Supplementary Table 1.** Overview of the included cohorts, with their in- and exclusion criteria.

|  | **N**  **(% of total)** | **Inclusion criteria** | **Exclusion criteria** |
| --- | --- | --- | --- |
| ***Cohorts*** |  |  |  |
| 1. Attention | 97 (7.7) | - MS diagnosis according to the 2010-McDonald criteria [1] - 18-68 years of age - Ability to safely undergo an MRI examination - Screening for motor and visual skills | - History or presence of drug abuse - Neurological (other than MS) and psychiatric diseases - Relapse and/or steroid treatment 4 weeks prior to examination |
| 2. Amsterdam MS cohort  *General MS cohort*  *Longstanding MS cohort* | 326 (26.9)  152/326 (46.6) | - MS diagnosis according to the 2010-McDonald criteria [1] - 18 years of age and older | - Neurological (other than MS) and psychiatric diseases - Relapse and/or steroid treatment 2 months prior to examination |
|  | 174/326 (53.3) | - MS diagnosis according to the 2010-McDonald criteria [1] - 18 years of age and older - Minimum disease duration of 10 years from onset | - Neurological (other than MS) and psychiatric diseases - Relapse and/or steroid treatment 6 weeks prior to examination |
| 3. MS&depression | 36 (3.0) | - MS diagnosis according to the 2010-McDonald criteria [1] - 18 years of age and older - Scoring >20 on the Beck Depression Inventory, 2^nd^ edition - Ability to safely undergo an MRI examination | - Elevated suicide risk - Psychotherapy - Using antidepressants for <6 weeks |
| 4. Fingolimod | 52 (4.3) | - MS diagnosis according to the 2010-McDonald criteria [1] - PwMS with RRMS - 18-65 years of age - Ability to safely undergo an MRI examination - Screening for motor and visual skills | - Neurological (other than MS) and psychiatric diseases - Relapse and/or steroid treatment 4 weeks prior to examination |

| 5. GABA & glutamate | 53 (4.4) | - MS diagnosis according to the 2017-McDonald criteria [2] - PwMS with RRMS or SPMS - 18-65 years of age - Ability to safely undergo an MRI examination - Screening for motor and visual skills | - History or presence of drug abuse - Neurological (other than MS) and psychiatric diseases - Relapse and/or steroid treatment 4 weeks prior to examination |
| --- | --- | --- | --- |
| 6. MS@Work | 287 (23.7) | - MS diagnosis according to the 2010-McDonald   criteria [1]   - 18 years of age and older - PwMS with RRMS - Screening for motor and visual skills - Employed (or within 3 years since their last employment) | - Inability to speak Dutch - Neurological (other than MS) and psychiatric diseases - Relapse and/or steroid treatment 4 weeks prior to examination - History or presence of drug abuse |
| 7. RemindMS | 104 (8.6) | - MS diagnosis according to the 2010-McDonald criteria[1] - 18-65 years of age - Scoring ≥23 on the Multiple Sclerosis Neuropsychological Questionnaire – Patient version (MSNQ-P) | - History/presence of psychosis and/or suicidal ideation - Inability to speak Dutch - Previous experience with the similar interventions - Physical or cognitive disabilities/ comorbidities/ treatments likely to cause interference |
| 8. SOMSCOG | 138 (11.4) | - MS diagnosis according to the 2017-McDonald criteria [2] |  |
| 9. Tecfidera | 65 (5.4) | - MS diagnosis according to the 2017-McDonald criteria [2] - PwMS with RRMS - 18-65 years of age - Ability to safely undergo an MRI examination - Screening for motor and visual skills | - History or presence of drug abuse - Neurological (other than MS) and psychiatric diseases - Relapse and/or steroid treatment 4 weeks prior to examination   Participation in other studies using cognitive or physical training programs |
| 10. Temprano | 58 (4.8) | - MS diagnosis according to the 2017-McDonald criteria, within one year [2] - PwMS with RRMS - 18-65 years of age - Sufficient Dutch proficiency - Ability to safely undergo an MRI examination | - History or presence of drug abuse - Neurological (other than MS) and psychiatric diseases - Relapse and/or steroid treatment 4 weeks prior to examination - Participation in other studies using cognitive or physical training programs |

*Abbrevations: MS = Multiple Sclerosis ; RRMS = Relapsing-Remitting MS.*

**Supplementary Table 2.** Overview of the included tests per cognitive domain, the corresponding test scores and the number of cohorts (ten in total) that included the specific test in their design.

|  | | **Neuropsychological test** | **Corresponding test scores** | **Number**  **of cohorts** |
| --- | --- | --- | --- | --- |
| ***Cognitive function*** | |  |  |  |
|  | Verbal memory | - California Verbal Learning Test – Version 2 | - Direct recall - Delayed recall - Recognition | 9 |
|  |  | - Selective Reminding Test | - Long-term storage 1 - Long-term retrieval sum - Short-term retrieval sum - Delayed recall | 1 |
|  | Visuospatial memory | - Location Learning Test | - Sum of displacement scores (five trials in total) | 4 |
|  |  | - Brief Visuospatial Memory Test – Revised | - Direct recall - Delayed recall - Recognition | 5 |
|  |  | - Spatial Recall Test | - Direct recall - Delayed recall | 1 |
|  | Information processing speed | - Symbol Digit Modalities Test | - Total of correct responses - reading subscale | 4 |
|  |  | - Letter Digit Substitution Test | - Total of correct responses - reading subscale | 6 |
|  | Attention | - Stroop Color-Word Test | - Time to complete card I - Time to complete card II | 9 |
|  |  | - Color-Word Interference Test |  | 1 |
|  | Executive functioning – Inhibition | - Stroop Color-Word Test | - Time to complete card III – (Time to complete card I + card II) | 9 |
|  |  | - Color-Word Interference Test |  | 1 |
|  | Executive functioning – Verbal fluency | - Controlled Oral Word Association Test | - Trial 1 (letter D) - Trial 2 (letter A) - Trial 3 (letter T) | 5 |
|  |  | - Word List Generation | - Trial 1 (animals) - Trial 2 (professions) - Trial 3 (m-words) | 5 |

**Supplementary Table 3.** An overview of the missing data and the demographics, clinical functioning, cognitive and psychological functioning for the non-imputed and the imputed data

|  | | **Non-imputed data** | | **Imputed data** |
| --- | --- | --- | --- | --- |
|  | | *n (% missing)* | description | *n* = 1213 |
| ***Demographics*** | | | | |
| Sex \| female (%) | | 1213 (0.0%) | 872 (71.9%) | 872 (71.9%) |
| Age | | 1191 (1.9%) | 45.5±10.7 | 45.4±10.7 |
| Education^a^ | | 1207 (0.5%) | 6.0 (5.0-6.0) | 6.0 (5.0-6.0) |
| ***Clinical functioning*** | | | | |
| MS type \| n (%) | | 1211 (0.2%) |  |  |
|  | RRMS |  | 1003 (82.7%) | 1005 (82.9%) |
|  | SPMS |  | 122 (10.1%) | 122 (10.1%) |
|  | PPMS |  | 63 (5.2%) | 63 (5.2%) |
|  | CIS |  | 5 (0.4%) | 5 (0.4%) |
|  | Unknown |  | 18 (1.5%) | 18 (1.5%) |
| Disease duration | | 1107 (8.5%) | 9.8±8.0 | 9.8±7.7 |
| EDSS | | 1139 (6.1%) | 3.3±1.7 | 3.3±1.6 |
| ***Cognitive functioning*** | | | | |
| Cognitive status \| CI (%) | | 1161 (4.3%) | 390 | 402 (33.1%) |
| Attention | | 1166 (3.9%) | -0.8±1.2 | -0.8±1.2 |
| Inhibition | | 1125 (7.3%) | -0.5±1.3 | -0.5±1.3 |
| IPS | | 1181 (2.6%) | -1.1±1.2 | -1.1±1.2 |
| Verbal fluency | | 1174 (3.2%) | -0.7±0.8 | -0.7±0.8 |
| Verbal memory | | 1178 (2.9%) | -0.7±1.2 | -0.7±1.2 |
| Visuospatial memory | | 1170 (3.6%) | -0.4±1.1 | -0.4±1.0 |
| ***PROMS*** | | | | |
| HADS-A | | 1115 (8.1%) | 6.2±3.8 | 6.3±3.7 |
| HADS-D | | 1114 (8.2%) | 4.6±3.7 | 4.6±3.6 |
| CIS20-R | | 723 (40.4%) | 82.5±24.0 | 79.7±21.1 |

Displayed are the mean ± standard deviation. ^a^ For ordinal or not-normally distributed variables, median and (interquartile range) are displayed. *Abbreviations: RRMS = Relapsing-Remitting MS; SPMS = Secondary Progressive MS; PPMS = Primary Progressive MS; CIS = Clinically Isolated Syndrome; EDSS = Expanded Disability Status Scale; CI = Cognitively Impaired; IPS = Information Processing Speed; PROMS = Patient Reported Outcome Measures; HADS-A = Hospital Anxiety and Depression Scale (HADS) – Anxiety subscale; HADS-D = HADS – Depression subscale; CIS20-R = Checklist Individual Strength 20 – Revised.*

**
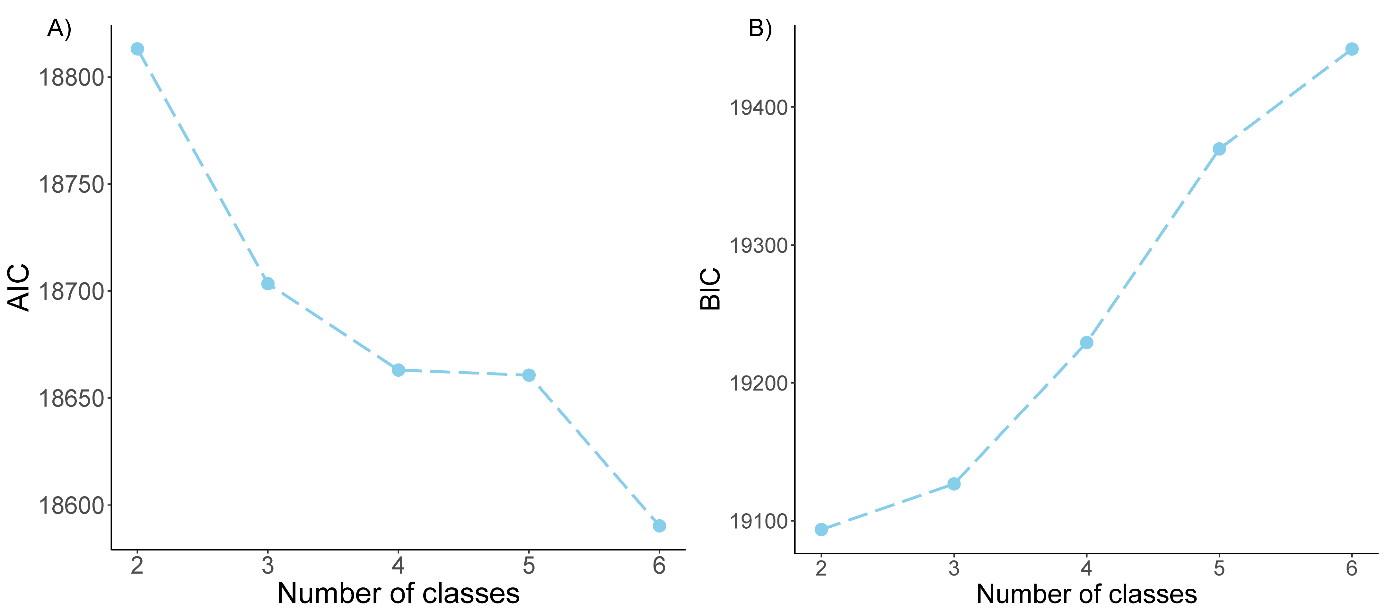
**

**Supplementary Figure 1.** A depiction of the fit of LPA with different number of classes (ranging from 2 up to 6). Panel A) shows the AIC (Akaike Information Criterion) for each number of classes. Panel B) shows the BIC (Bayesian Information Criterion) for each number of classes.

**
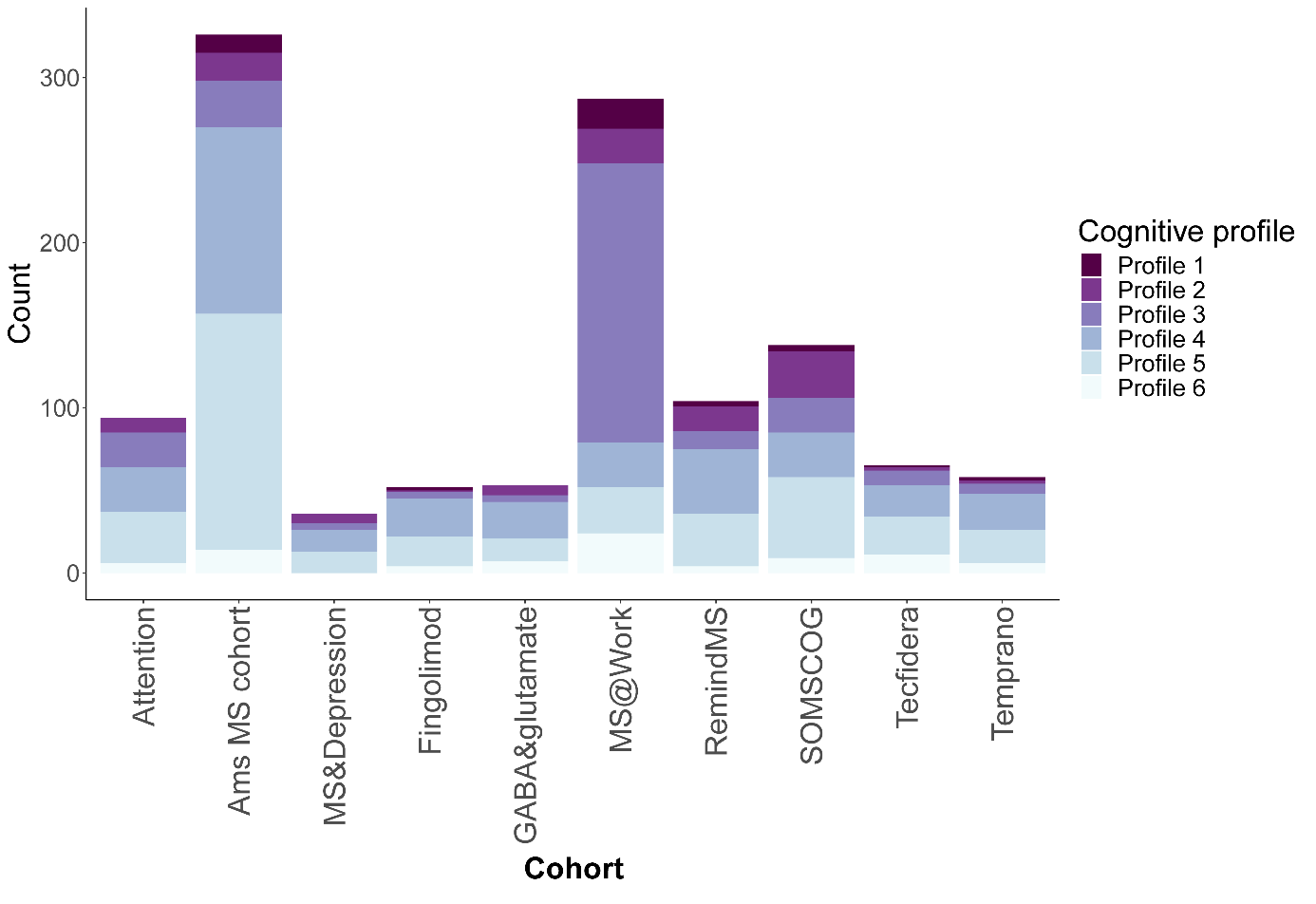
**

**Supplementary Figure 2.** Depiction of the count of PwMS per cohort belonging to a certain cognitive profile. *Abbreviations: Ams = Amsterdam.*

**Supplementary Table 4.** An overview of the demographics, clinical functioning, cognitive functioning and PROMS per cognitive profile on non-imputed data

|  | | **Profile 1**  (*n* = 85) | **Profile 2**  (*n* = 277) | **Profile 3**  (*n* = 41) | **Profile 4**  (*n* = 332) | **Profile 5**  (*n* = 371) | **Profile 6**  (*n* = 107) | ***p*-value** |
| --- | --- | --- | --- | --- | --- | --- | --- | --- |
| ***Demographics*** | | | | | | | | |
| Sex \| female (%) | | 66 (77.6%) | 214 (77.3%) | 32 (78.0%) | 217 (65.4%) | 267 (72.0%) | 76 (71.0%) | **0.022** |
| Age | | 43.9±10.5 | 43.3±9.8 | 47.8±12.5 | 45.9±10.8 | 45.9±10.9 | 48.8±11.1 | **<.001*** |
| Education^a^ | | 6.0 (5.0-6.0) | 6.0 (5.0-6.0) | 5.0 (5.0-6.0) | 6.0 (5.0-6.0) | 6.0 (5.0-6.0) | 6.0 (5.0-6.0) | **0.002** |
| ***Clinical functioning*** | | | | | | | | |
| MS type \| n (%) | |  |  |  |  |  |  | **<.001*** |
|  | RRMS | 76 (89.4%) | 258 (93.1%) | 36 (87.8%) | 267 (80.4%) | 290 (78.2%) | 76 (71.0%) |  |
|  | SPMS | 8 (9.4%) | 10 (3.6%) | 4 (9.8%) | 33 (9.9%) | 48 (12.9%) | 19 (17.8%) |  |
|  | PPMS | 1 (1.2%) | 5 (1.8%) | 0 (0.0%) | 22 (6.6%) | 28 (7.5%) | 7 (6.5%) |  |
|  | CIS | 0 (0.0%) | 1 (0.4%) | 0 (0.0%) | 3 (0.9%) | 0 (0.0%) | 1 (0.9%) |  |
|  | Unknown | 0 (0.0%) | 2 (0.7%) | 0 (0.0%) | 7 (2.1%) | 5 (1.3%) | 4 (3.7%) |  |
| Disease duration | | 8.8±7.7 | 8.2±6.8 | 7.5±6.3 | 10.2±8.2 | 10.2±8.1 | 13.3±9.5 | **<.001*** |
| EDSS^a^ | | 3.0 (2.0-4.5) | 2.5 (1.5-3.5) | 3.0 (2.0-5.0) | 3.5 (2.5-4.0) | 3.5 (2.5-4.0) | 4.0 (3.0-5.5) | **<.001*** |
| ***Cognitive functioning*** | | | | | | | | |
| Cognitive status \| CI (%) | | 10 (11.8%) | 40 (14.4%) | 7 (17.1%) | 106 (31.9%) | 127 (34.2%) | 100 (92.5%) | **<.001*** |
| Attention | | -0.4±0.7 | -0.1±0.7 | -0.4±0.3 | -0.4±0.9 | -1.1±1.1 | -2.6±1.6 | **<.001*** |
| Inhibition | | 0.2±1.1 | 0.2±0.8 | -0.7±0.7 | -0.3±1.2 | -0.9±1.1 | -1.8±2.1 | **<.001*** |
| IPS | | -0.5±1.4 | -1.0±0.8 | -1.2±0.8 | -0.9±1.1 | -1.2±1.3 | -2.4±1.1 | **<.001*** |
| Verbal fluency | | -0.3±0.8 | -0.3±1.0 | -0.8±0.5 | -0.5±0.6 | -1.1±0.4 | -1.2±0.8 | **<.001*** |
| Verbal memory | | -0.3±0.7 | -0.8±1.2 | -0.8±0.6 | -0.6±1.2 | -0.3±0.8 | -2.3±1.3 | **<.001*** |
| Visuospatial memory | | -0.5±0.4 | 0.1±0.3 | -1.0±1.1 | -0.9±1.3 | -0.9±0.8 | -1.4±1.2 | **<.001*** |
| ***PROMS*** | | | | | | | | |
| HADS-A | | 5.5±3.5 | 6.4±3.5 | 6.5±4.1 | 5.9±3.7 | 6.3±4.0 | 7.1±4.5 | 0.38 |
| HADS-D | | 3.6±3.5 | 4.1±3.2 | 5.6±4.2 | 4.3±3.5 | 5.1±4.1 | 5.6±.3.5 | **<.001*** |
| CIS20-R | | 72.7±25.6 | 82.4±22.3 | 86.8±22.4 | 79.4±24.1 | 84.9±23.7 | 89.4±23.2 | **<.001*** |

Displayed are the mean ± standard deviation. ^a^ For ordinal or not-normally distributed variables, median and (interquartile range) are displayed. ^b^ corrected *p*-values. * Significant between all profiles, at an α-level of .05, after correcting for multiple comparisons using Bonferroni. *Abbreviations: RRMS = Relapsing-Remitting MS; SPMS = Secondary Progressive MS; PPMS = Primary Progressive MS; CIS = Clinically Isolated Syndrome; EDSS = Expanded Disability Status Scale; CI = Cognitively Impaired; IPS = Information Processing Speed; PROMS = Patient Reported Outcome Measures; HADS-A = Hospital Anxiety and Depression Scale (HADS) – Anxiety subscale; HADS-D = HADS – Depression subscale; CIS20-R = Checklist Individual Strength 20 – Revised.*

***
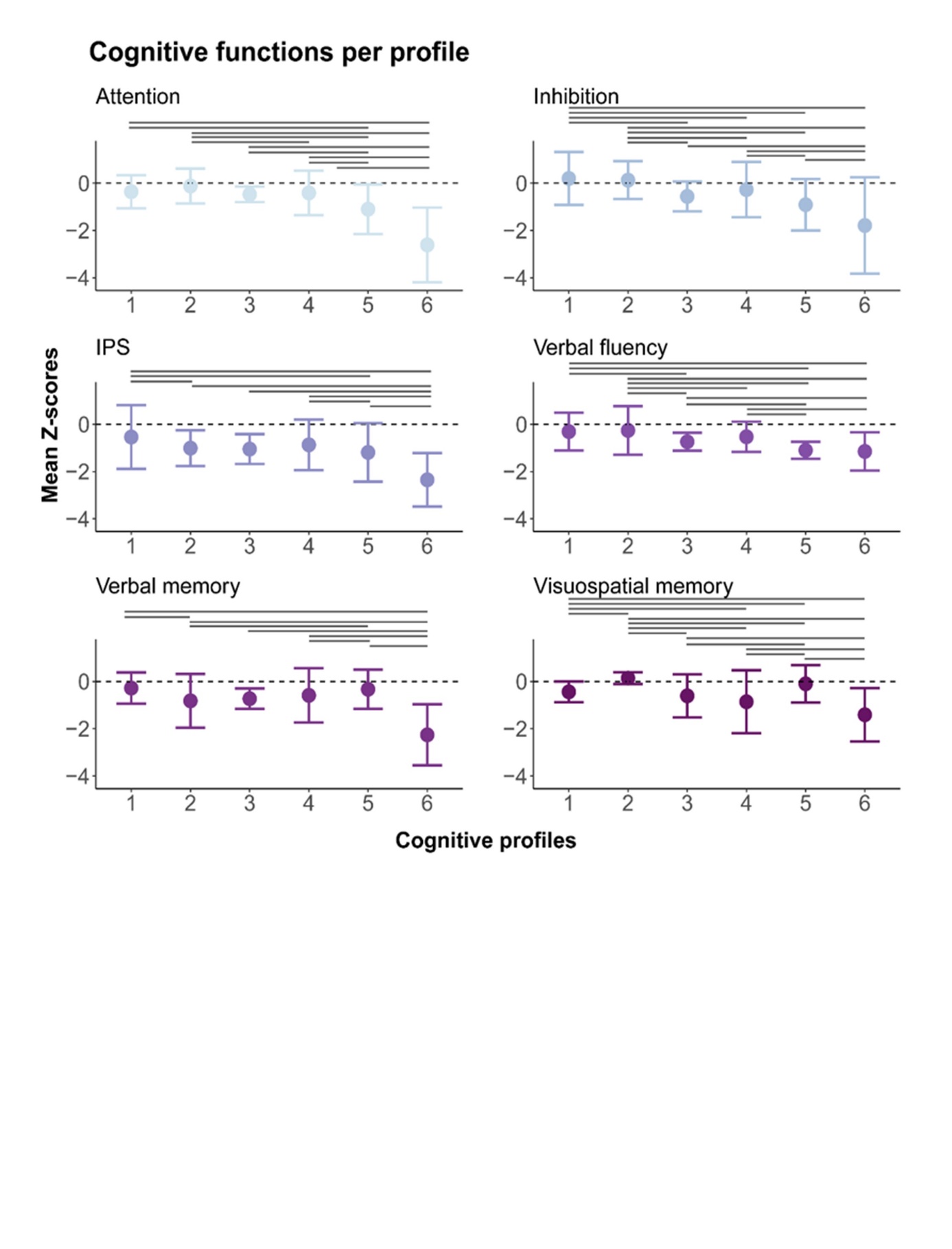
***

**Supplementary Figure 3.** Significant post-hoc differences (indicated with a black stripe) for each cognitive function (mean Z-scores, on the y-axis) between profiles (number on the x-axis). *Abbreviations: CP = Cognitively Preserved; CI = Cognitively Impaired; IPS = Information Processing Speed.*

**Supplementary Table 5.** Post-hoc differences between profiles for cognitive status and the cognitive functions

|  | 1-p2 | p1-p3 | p1-p4 | p1-p5 | p1-p6 | p2-p3 | p2-p4 | p2-p5 | p2-p6 | p3-p4 | p3-p5 | p3-p6 | p4-p5 | p4-p6 | p5-p6 |
| --- | --- | --- | --- | --- | --- | --- | --- | --- | --- | --- | --- | --- | --- | --- | --- |
| ***CP vs. CI*** | .532 | .244 | **<.001** | **<.001** | **<.001** | .397 | **<.001** | **<.001** | **<.001** | .071 | .042 | **<.001** | 0.601 | **<.001** | **<.001** |
| ***Cognitive functions*** | |  |  |  |  |  |  |  |  |  |  |  |  |  |  |
| Attention | .453 | .113 | .052 | **<.001** | **<.001** | .194 | **<.001** | **<.001** | **<.001** | .012 | **<.001** | **<.001** | **<.001** | **<.001** | **<.001** |
| Inhibition | .004 | .777 | .07 | **<.001** | **<.001** | .004 | **<.001** | **<.001** | **<.001** | .297 | .013 | **<.001** | **<.001** | **<.001** | **<.001** |
| IPS | .385 | .716 | .248 | .012 | **<.001** | .303 | .673 | **0.01** | **<.001** | .219 | .029 | **<.001** | 0.023 | **<.001** | **<.001** |
| Verbal fluency | .271 | .081 | .887 | .073 | **<.001** | .024 | .047 | .246 | **<.001** | .089 | .010 | **<.001** | **.001** | **<.001** | **<.001** |
| Verbal memory | **<.001** | .350 | **<.001** | .058 | **<.001** | .006 | .234 | **<.001** | **<.001** | .021 | .586 | **<.001** | **<.001** | **<.001** | **<.001** |
| Visuospatial memory | .071 | **<.001** | **<.001** | .262 | **<.001** | **<.001** | **<.001** | **.002** | **<.001** | .037 | **<.001** | **.003** | **<.001** | .064 | **<.001** |

Displayed are the uncorrected *p*-values. In bold are the comparisons between profiles that remained significant after Bonferroni correction for multiple comparisons (α-level of .05 divided by 15, leading to a new α-level of .003). *Abbreviations: CP = Cognitively Preserved; CI = Cognitively Impaired; IPS = Information Processing Speed; p = profile.*

**Supplementary Table 6.** An overview of the demographics, clinical functioning, cognitive functioning and PROMS of the train and test datasets.

|  | **Train**  (*n* = 729) | **Test**  (*n* = 484) | ***p*-value** |
| --- | --- | --- | --- |
| ***Demographics*** | | | |
| Sex (f:m) | 524 : 205 (71.9%f) | 348 : 136 (71.9%f) | 0.993 |
| Age | 45.36±10.70 | 45.45±10.64 | 0.886 |
| Education^a^ | 6.0 (5.0-6.0) | 6.0 (5.0-6.0) | 0.533 |
| ***Clinical functioning*** | | | |
| MS type  (PPMS/SPMS/RRMS/CIS/Unknown) | 40/79/598/2/10 | 23/43/407/3/8 | 0.664 |
| Disease duration | 10.06±7.88 | 9.50±7.36 | 0.212 |
| EDSS | 3.30±1.66 | 3.34±1.61 | 0.694 |
| ***Cognitive functioning*** | | | |
| Attention | -0.77±1.21 | -0.71±1.18 | 0.363 |
| Inhibition | -0.53±1.31 | -0.42±1.29 | 0.152 |
| IPS | -1.15±1.18 | -1.06±1.14 | 0.175 |
| Verbal fluency | -0.72±0.79 | -0.64±0.79 | 0.105 |
| Verbal memory | -0.72±1.17 | -0.66±1.12 | 0.404 |
| Visuospatial memory | -0.44±1.09 | -0.36±0.97 | 0.166 |
| ***PROMS*** | | | |
| HADS-A | 6.24±3.67 | 6.25±3.73 | 0.988 |
| HADS-D | 4.62±3.58 | 4.67±.3.56 | 0.818 |
| CIS20-R | 79.39±20.91 | 80.18±21.38 | 0.522 |

*Abbreviations: f = female; m = male; PP = Primary Progressive; SP = Secondary Progressive; RR = Relapsing-Remitting; CIS = Clinically Isolated Syndrome; EDSS = Expanded Disability Status Scale; IPS = Information Processing Speed; PROMS = Patient Reported Outcome Measures; HADS-A = Hospital Anxiety and Depression Scale (HADS) – Anxiety subscale; HADS-D = HADS – Depression subscale; CIS20-R = Checklist Individual Strength 20 – Revised.*

**Supplementary Table 7.** An overview of the demographics, clinical functioning, cognitive functioning and PROMS per cognitive status

|  | **Cognitively preserved**  (*n* = 811) | **Cognitively impaired**  (*n* = 402) | ***p*-value** |
| --- | --- | --- | --- |
| ***Demographics*** | | | |
| Sex (f:m) | 612 : 199 (75.5%f) | 260 : 142 (64.7%f) | **<.001** |
| Age | 44.23±10.45 | 47.74±10.72 | **<.001** |
| Education^a^ | 6.0 (5.0-6.0) | 6.0 (5.0-6.0) | **<.001** |
| ***Clinical functioning*** | | | |
| MS type  (PPMS/SPMS/RRMS/CIS/Unknown) | 34/56/710/3/8 | 29/66/295/2/10 | **<.001** |
| Disease duration | 8.88±7.11 | 11.77±8.40 | **<.001** |
| EDSS | 3.00±1.57 | 3.95±1.59 | **<.001** |
| ***Cognitive functioning*** | | | |
| Attention | -0.26±0.83 | -1.74±1.22 | **<.001** |
| Inhibition | -0.08±0.99 | -1.31±1.46 | **<.001** |
| IPS | -0.64±0.92 | -2.07±1.00 | **<.001** |
| Verbal fluency | -0.48±0.77 | -1.12±0.66 | **<.001** |
| Verbal memory | -0.33±0.94 | -1.43±1.19 | **<.001** |
| Visuospatial memory | -0.09±0.84 | -1.06±1.11 | **<.001** |
| ***PROMS*** | | | |
| HADS-A | 6.01±3.54 | 6.72±3.93 | **0.002** |
| HADS-D | 4.18±3.40 | 5.56±.3.71 | **<.001** |
| CIS20-R | 76.43±21.00 | 86.31±19.71 | **<.001** |

*Abbreviations: f = female; m = male; PP = Primary Progressive; SP = Secondary Progressive; RR = Relapsing-Remitting; CIS = Clinically Isolated Syndrome; EDSS = Expanded Disability Status Scale; IPS = Information Processing Speed; PROMS = Patient Reported Outcome Measures; HADS-A = Hospital Anxiety and Depression Scale (HADS) – Anxiety subscale; HADS-D = HADS – Depression subscale; CIS20-R = Checklist Individual Strength 20 – Revised.*

**Supplementary Table 8.** List of booster hyper-parameters of gradient boosting decision trees after grid search

|  | | **Outcome:**  **cognitive profiles** | **Outcome:**  **cognitive status** |
| --- | --- | --- | --- |
| ***List of parameters*** | |  |  |
|  | Eta | 0.01 | 0.01 |
|  | Gamma | 6 | 8 |
|  | Max_depth | 5 | 8 |
|  | Subsample | 0.7 | 0.7 |
|  | Colsample_bytree | 0.8 | 0.8 |
|  | Lambda | 2 | 3 |

**Supplemental References**

[1] C. H. Polman *et al.*, "Diagnostic criteria for multiple sclerosis: 2010 revisions to the McDonald criteria," *Annals of neurology,* vol. 69, no. 2, pp. 292-302, 2011. [Online]. Available: <https://onlinelibrary.wiley.com/doi/pdf/10.1002/ana.22366>.

[2] A. J. Thompson *et al.*, "Diagnosis of multiple sclerosis: 2017 revisions of the McDonald criteria," *The Lancet Neurology,* vol. 17, no. 2, pp. 162-173, 2018.
